# Genome-wide association mapping for dominance effects in female fertility using real and simulated data from Danish Holstein cattle

**DOI:** 10.1038/s41598-020-59788-5

**Published:** 2020-02-19

**Authors:** Xiaowei Mao, Goutam Sahana, Anna Maria Johansson, Aoxing Liu, Ahmed Ismael, Peter Løvendahl, Dirk-Jan De Koning, Bernt Guldbrandtsen

**Affiliations:** 1 Key Laboratory of Vertebrate Evolution and Human Origins, Institute of Vertebrate Paleontology and Paleoanthropology, Chinese Academy of Sciences, Beijing, 100044 China; 2 CAS Center for Excellence in Life and Paleoenvironment, Beijing, 100044 China; 3 Center for Quantitative Genetics and Genomics, Department of Molecular Biology and Genetics, Aarhus University, 8830 Tjele, Denmark; 4 Department of Animal Breeding and Genetics, Swedish University of Agricultural Sciences, 75007 Uppsala, Sweden; 5 Laboratory of Animal Genetics, Breeding and Reproduction, College of Animal Science and Technology, China Agricultural University, 100193 Beijing, China; 6 Scion, 49 Sala St, Rotorua, New Zealand

**Keywords:** Data mining, Genome-wide association studies, Quantitative trait

## Abstract

Exploring dominance variance and loci contributing to dominance variation is important to understand the genetic architecture behind quantitative traits. The objectives of this study were i) to estimate dominance variances, ii) to detect quantitative trait loci (QTL) with dominant effects, and iii) to evaluate the power and the precision of identifying loci with dominance effect through post-hoc simulations, with applications for female fertility in Danish Holstein cattle. The female fertility records analyzed were number of inseminations (NINS), days from calving to first insemination (ICF), and days from the first to last insemination (IFL), covering both abilities to recycle and to get pregnant in the female reproductive cycle. There were 3,040 heifers and 4,483 cows with both female fertility records and Illumina BovineSNP50 BeadChip genotypes (35,391 single nucleotide polymorphisms (SNP) after quality control). Genomic best linear unbiased prediction (BLUP) models were used to estimate additive and dominance genetic variances. Linear mixed models were used for association analyses. A post-hoc simulation study was performed using genotyped heifers’ data. In heifers, estimates of dominance genetic variances for female fertility traits were larger than additive genetic variances, but had large standard errors. The variance components for fertility traits in cows could not be estimated due to non-convergence of the statistical model. In total, five QTL located on chromosomes 9, 11 (2 QTL), 19, and 28 were identified and all of them showed both additive and dominance genetic effects. Among them, the SNP rs29018921 on chromosome 9 is close to a previously identified QTL in Nordic Holstein for interval between first and last insemination. This SNP is located in the 3’ untranslated region of gene peptidylprolyl isomerase like 4 (*PPIL4*), which was shown to be associated with milk production traits in US Holstein cattle but not known for fertility-related functions. Simulations indicated that the current sample size had limited power to detect QTL with dominance effects for female fertility probably due to low QTL variance. More females need to be genotyped to achieve reliable mapping of QTL with dominance effects for female fertility.

## Introduction

Intensive selection on milk yield in dairy cattle has led to a decline in female fertility, due to unfavorable genetic correlations between milk yield and female fertility^1^. Declining female fertility increases the costs for dairy farm management due to extra inseminations, veterinary treatments, and involuntary replacements^2^. Moreover, poor female fertility has been showed to be genetically correlated with increased methane production and negative climate impact^3^. Female fertility in dairy cattle can be generally divided into two component^4,5^. The first component is the ability to return to cycling and to go into heat after calving, which can be measured by the time interval from calving to first insemination. The second component is the ability to conceive and become pregnant, which can be measured by the time interval between the first and last inseminations or the number of inseminations per conception.

Female fertility traits in cattle have low heritabilities (generally less than 5%), which has been well documented in various Holstein populations^6–8^. Apart from the genetic and environmental factors, management decisions can also influence the fertility phenotypes (e.g., voluntary waiting period)^6,7^. It has been reported that higher estimated heritabilities were achieved using activity measurements to indicate estrus in dairy cows compared with using traditional measurements calculating from calving and insemination records^5,9^. Another reason could be that the non-additive part of the genetic variation was often not considered due to the application of additive-only models^10^. Dominance effects are an important part of non-additive genetic effects. They represent the effect of interactions between alleles at the same locus^11^. Dominance genetic effects have been reported to make a substantial contribution (up to 14% of phenotypic variance) to the genetic variation in yearling weight in cattle^12^. Thus, understanding the genetic architecture in the presence of dominance effects is helpful for planning breeding strategies and increasing genetic gain. For example, dominance effects can be utilized by designing mating schemes that optimize favorable allele combinations, especially for crossbreeding which benefit from heterosis^13^.

The ability to estimate dominance variance using pedigree-based approach is limited by the requirement for data with large full-sib families and the high computational complexity involved^14^. The availability of high throughput genotyping technology enables the investigation of dominance effect using genomic information. From the computational perspective, the investigation of dominance effect using a genomic-based approach is much simpler than using pedigree-based approaches. This is due to that heterozygotes and homozygotes can be directly distinguished by genomic information. For milk yield in dairy cattle, the dominance variance accounted for up to 7% of total phenotypic variance, while the additive variance accounted for up to 30% of total phenotypic variance^14^. Furthermore, including dominance effects in the analyses can improve the fitness of models and the reliability of genomic predictions^15^.

In recent years, a number of studies have identified recessive alleles, including embryonic recessive lethal allele segregating in modern cattle populations^16,17^. By definition, recessive alleles have large dominance effects. One consequence of the existences of recessive alleles is the genetic variation in female fertility, as females carrying recessive lethal alleles will lose embryos that become homozygous for one of these alleles. In addition, female fertility traits tend to suffer from inbreeding depression^18^. This also suggests substantial dominance effects acting on female fertility. The estimation of dominance variance components for female fertility traits using pedigree-based approach has previously been reported^19,20^. In US Holstein, the broad sense heritability (2.2% to 6.6%) was reported to be at least twice as large as the narrow sense heritability for female fertility traits^19^. In Austrian Simmental and Brown Swiss dairy cattle, similar values for additive (1.00%) and dominance (0.32% to 1.36%) variances were observed for female fertility traits^20^. To our knowledge, however, the estimation of the dominance genetic variance using genomic-based approach has not previously been applied on female fertility in dairy cattle.

Genome-wide association studies (GWAS) have become a useful tool to reveal the genetic architecture for complex traits^21^. However, few GWAS for dominance effects have been performed in dairy cattle, despite large dominance genetic variances for some traits^15^. In contrast to GWAS for additive effects which were commonly performed on estimated breeding values, the detection of dominance effects requires genotyped animals having their own phenotypic records. Recently, the accumulation of cows with both phenotypes and genotypes has enabled the investigation of dominance genetic effects by GWAS. For example, suggestive QTL for dominance effects located on chromosome 2, 3, 5, 26 and 27 for milk yield and located on chromosome 1, 2, 3, 7, 23, 25 and 28 for female fertility have been reported in dairy cattle^22^. The statistical power for detecting QTL with dominance genetic effects is expected to be low, due to reliability of estimated genetic value of cows is much lower than those of proven bulls.

In Danish Holsteins, large numbers of heifers and cows have been recently genotyped with low-density SNP chips for the purpose of genomic evaluation. Simultaneously, large amounts of phenotypic information are available for farm management and for breeding value estimation. These data open the opportunity for the estimation of dominance variances and the detection of QTL with dominance genetic effects in dairy cattle, in contrast to pseudo phenotypes such as estimated breeding values or de-regressed proofs where dominance genetic effects are not included.

The objectives of this study were to 1) estimate additive and dominance variances for female fertility traits in Danish Holsteins; 2) detect QTL with additive and dominance effects; 3) evaluate the power and the precision using simulation when detecting QTL with dominance effects for our study design.

## Materials and Methods

### Ethics statement

All phenotypic and genomic data were recorded for the purpose of routine dairy cattle managements and genomic evaluations previously, followed guidelines and regulations by the Danish Animal Experiments Inspectorate. Ethics review and approval was not required for this study, because no additional animal handling or experiment was performed specifically for this study.

### Animals and phenotypes

Insemination and calving records of Danish Holsteins born from 2004 to 2012 in 5,248 herds were used in our analyses. The raw data included insemination records for 738,049 heifers and 974,715 cows in lactations 1–3. The performances of heifers and cows were considered as different traits. The traits analyzed were number of inseminations (NINS), days from calving to first insemination (ICF), and days from the first to last insemination (IFL), which covered both abilities to recycle and to get pregnant in the female reproductive cycle. The ICF was only available in cows, while IFL and NINS were available in both heifers and cows. For IFL and NINS, a suffixes h (for heifers) or c (for cows) was attached to the trait abbreviations. The raw data were edited according to standard procedures of Nordic Cattle Genetic Evaluation (https://www.nordicebv.info/health/) with slight modifications. Only records for heifers with age at first insemination older than 270 days and younger than 900 days, NINSh less than 8 and IFLh less than 365 days were kept. Only records of cows with age at first calving older than 550 days and younger than 1100 days, NINSc smaller than 8, ICF less than 230 days, and IFLc less than 365 days were kept. After editing, there were 714,759 heifers and 905,447 cows left for further analyses. Approximately 15% of them had no confirmed successful insemination. Thus, the last insemination was taken as an unsuccessful insemination, and the corresponding records were considered as censored records. A penalty of 21 days was added to censored IFL and 1 “count” was added to censored NINS^6^.

### Genotypes

Among the animals with phenotypic records, 3,040 heifers and 4,483 cows were genotyped with Illumina BovineSNP50 BeadChip version 1 or 2 (Illumina Inc., San Diego, CA)^23^. A total of 55,298 single nucleotide polymorphisms (SNPs) on 29 *Bos taurus* autosomes and the X chromosome were available. The numbers of SNPs per chromosome ranged from 894 on the X chromosome to 2,502 on the chromosome 1. A routine quality control was conducted using the software Plink^24^ to remove SNPs with minor allele frequencies <5%, call rate <90% or a significant deviation from the Hardy–Weinberg proportion (*P*-value < 10^−5^ for *χ*^2^ test with one degree of freedom). After quality control, the number of SNPs remaining was 35,391. The UMD v3.1 assembly^25^ was used as the reference for genomic position of the SNPs.

### Pre-correction of fixed effects for phenotypes

The original phenotypes were pre-corrected for fixed effects before estimation of variance components and association analyses. In this way, the fixed effects were estimated more accurately using all animals with phenotypes (714,759 heifers and 905,447 cows) rather than just using the genotyped subset (3,040 heifers and 4,483 cows). A pedigree-based sire model applied in the DMU software^26^ was used to pre-correct phenotypes for heifers and cows separately.

The model for heifers was:

1 $${\boldsymbol{y}}={\boldsymbol{Xb}}+{{\boldsymbol{Z}}}_{{\boldsymbol{s}}}{\boldsymbol{s}}+{\boldsymbol{e}}$$

The model for cows was:

2 $${\boldsymbol{y}}={\boldsymbol{Xb}}+{{\boldsymbol{Z}}}_{{\boldsymbol{s}}}{\boldsymbol{s}}+{\boldsymbol{Zpe}}+{\boldsymbol{e}}$$

where ${\boldsymbol{y}}$ was the vector of the observations of a particular fertility trait, ***b*** was the vector of fixed effects including herd, year-month of first insemination, age at the first insemination (in days, only for heifers), age at the first calving (in days, only for cows) and parities (only for cows), ${\boldsymbol{X}}$ was the incidence matrix relating fixed effects to individual observations, and ${{\boldsymbol{Z}}}_{{\boldsymbol{s}}}$ and ${\boldsymbol{Z}}$ were incidence matrices relating the vector of random sire effects ${\boldsymbol{s}}$ and the vector of random permanent environmental effects ${\boldsymbol{pe}}$ to ${\boldsymbol{y}}$, and ${\boldsymbol{e}}$ was the vector of random residuals. The values ${\boldsymbol{s}}$, ${\boldsymbol{pe}}$, and ${\boldsymbol{e}}$ were assumed to follow normal distributions with ${\boldsymbol{s}} \sim {\boldsymbol{N}}({\bf{0}},\,{\sigma }_{s}^{2}{{\boldsymbol{A}}}_{{\boldsymbol{s}}})$, ${\boldsymbol{pe}} \sim {\boldsymbol{N}}({\bf{0}},\,{\sigma }_{pe}^{2}{\boldsymbol{I}})$ and ${\boldsymbol{e}} \sim {\boldsymbol{N}}({\bf{0}},\,{\sigma }_{e}^{2}{\boldsymbol{I}})$, where ${{\boldsymbol{A}}}_{{\boldsymbol{s}}}$ was the pedigree relationship matrix for the sires, ${\boldsymbol{I}}$ was an identity matrix, ${\sigma }_{s}^{2}$ was the variance of sire effects, ${\sigma }_{pe}^{2\,}$ was the variance of permanent environmental effects, and ${\sigma }_{e}^{2\,}\,$ was the residual variance. Then, pre-corrected phenotypes ${{\boldsymbol{y}}}_{{\boldsymbol{c}}}$ for heifers were calculated as ${{\boldsymbol{y}}}_{{\boldsymbol{hc}}}={{\boldsymbol{Z}}}_{{\boldsymbol{s}}}{\boldsymbol{s}}+{\boldsymbol{e}}$, and for cows as ${{\boldsymbol{y}}}_{{\boldsymbol{cc}}}={{\boldsymbol{Z}}}_{{\boldsymbol{s}}}{\boldsymbol{s}}+\mathop{\sum }\limits_{k=1}^{n}{\boldsymbol{e}}/n$, where $n$ was the number of parities. The reliabilities for ${{\boldsymbol{y}}}_{{\boldsymbol{cc}}}$ were estimated by ${r}_{cc}^{2}=\frac{{\rm{n}}{h}_{cc}^{2}}{(n-1){h}_{cc}^{2}+1}$, where ${h}_{cc}^{2}$ was the heritability for the traits. Thus, the range of reliabilities for ${{\boldsymbol{y}}}_{{\boldsymbol{cc}}}$ were from 0.03 to 0.08 for NINS, from 0.04 to 0.11 for ICF, and from 0.02 to 0.06 for IFL.

### Estimation of additive and dominance genetic variances

Genomic best linear unbiased prediction (BLUP) model was applied to estimate additive and dominance genetic variances using pre-corrected phenotypes. Both additive and dominance genetic relationship matrices were constructed from the genome-wide marker data. Compared with pedigree based relationship matrices, marker-based relationship matrices include both genetic links through unknown common ancestors and the Mendelian sample variation. The genomic BLUP model was:

3 $${{\boldsymbol{y}}}_{{\boldsymbol{c}}}=1\mu +{{\boldsymbol{Z}}}_{{\boldsymbol{a}}}{\boldsymbol{a}}+{{\boldsymbol{Z}}}_{{\boldsymbol{d}}}{\boldsymbol{d}}+{\boldsymbol{e}}$$

where ${{\boldsymbol{y}}}_{{\boldsymbol{c}}}$ was the vector of pre-corrected phenotypes for heifers (${{\boldsymbol{y}}}_{{\boldsymbol{hc}}}$) and cows (${{\boldsymbol{y}}}_{{\boldsymbol{cc}}}$), **1** was the vector of ones, $\mu$ was the general mean; ***a*** was the vector of additive genetic effects, and ***d*** was the vector of dominance effects. The *a* and *d* were assumed to follow normal distributions, ${\boldsymbol{a}} \sim {\boldsymbol{N}}({\bf{0}},\,{\sigma }_{a}^{2}{\boldsymbol{G}})$ and ${\boldsymbol{d}} \sim {\boldsymbol{N}}({\bf{0}},\,{\sigma }_{d}^{2}{\boldsymbol{D}})$, where *G* was the genomic additive relationship matrix, ${\boldsymbol{D}}$ was the genomic dominance relationship matrix, ${\sigma }_{a}^{2}$ was the additive genetic variance, and ${\sigma }_{d}^{2}$ was the dominance genetic variance. The **G** and **D** were built from genotypes, but excluding SNPs on the X chromosome^10^. In brief, the additive genomic relationship matrix was constructed following previous studies^27,28^, the dominance genomic relationship matrix $D=\frac{H{\boldsymbol{H}}{\boldsymbol{{\prime} }}}{{\sum }_{j=1}^{k}2{p}_{j}{q}_{j}(1-2{p}_{j}{q}_{j})}$, where $k$ was the total number of SNPs, ${q}_{j}$ and ${p}_{j}$ were the frequencies of first and second allele at locus $j$, respectively. Element $(i,\,j)$ of ${\boldsymbol{H}}$ was $1-2{p}_{j}{q}_{j}$ if individual $i$ was heterozygous at locus $j$ and $-2{p}_{j}{q}_{j}$ otherwise. The ${{\boldsymbol{Z}}}_{{\boldsymbol{a}}}$ and ${{\boldsymbol{Z}}}_{{\boldsymbol{d}}}$ were incidence matrices relating elements of the vector of additive and dominance effects to ${{\boldsymbol{y}}}_{{\boldsymbol{c}}}$. It was assumed ${\boldsymbol{e}} \sim {\bf{N}}({\bf{0}},{\sigma }_{e}^{2}{\boldsymbol{W}}$), where ${\boldsymbol{W}}$ were an identity matrix for heifer traits and a diagonal matrix with the elements ${W}_{ii}=1/{w}_{i}^{\ast }$ for cows traits, in which ${w}_{i}^{\ast }$ was the standardized weight of ${{\boldsymbol{y}}}_{{\boldsymbol{cc}}}$. The ${w}_{i}^{\ast }$ were defined by first calculating the weight of ${{\boldsymbol{y}}}_{{\boldsymbol{cc}}}$ as $w=\frac{{r}_{cc}^{2}}{1-{r}_{cc}^{2}}$, and then $w$ was standardized to ${w}_{i}^{\ast }$. The average information-restricted maximum likelihood implemented in the DMU software^26^ was used to estimate additive and dominance genetic variances. Asymptotic standard errors of variance component estimates were obtained from the average information matrix. The standard errors of heritabilities were estimated using an expansion of the Taylor series^29^.

### Association analyses

A mixed model analysis^30^ implemented in the DMU software^26^ was used to test associations between SNPs and pre-corrected phenotypes for each trait. The SNPs were fitted into the model one by one:

4 $${{\boldsymbol{y}}}_{{\boldsymbol{c}}}={\boldsymbol{M}}{{\boldsymbol{g}}}_{}+{\boldsymbol{Zu}}+{\boldsymbol{e}}$$

where ${{\boldsymbol{y}}}_{{\boldsymbol{c}}}$ was the vector of pre-corrected phenotypes for heifers (${{\boldsymbol{y}}}_{{\boldsymbol{hc}}}$) and cows (${{\boldsymbol{y}}}_{{\boldsymbol{cc}}}$), ${\boldsymbol{M}}$ was the incidence matrix for the genotypes and ${\boldsymbol{g}}$ was the vector of three SNP genotype (AA, Aa and aa) as fixed effects, and ${\boldsymbol{Z}}$ was the incidence matrix relating observations to elements of the vector of polygenic effects ${\boldsymbol{u}}$. The values of ${\boldsymbol{u}}$ were assumed to follow a normal distribution ${\boldsymbol{u}} \sim {\boldsymbol{N}}({\bf{0}},{\sigma }_{g}^{2}{\boldsymbol{A}})$, where ${\boldsymbol{A}}$ was the pedigree relationship matrix for the heifers or cows, ${\sigma }_{g}^{2}$ was the variance of polygenic effects. The pedigree was traced as far back in time as possible (28 generations), to capture population stratification and family structure well. It was assumed, that ${\boldsymbol{e}} \sim {\boldsymbol{N}}({\bf{0}},{\sigma }_{e}^{2}{\boldsymbol{W}})$, where ${\sigma }_{e}^{2}$ and ${\boldsymbol{W}}$ were the same as those defined in the genomic BLUP model.

### Significance test for additive and dominance effects

The additive and dominance effects of a SNP were tested in a two-step procedure^31^. First, the effects of the genotypes were tested against the null hypothesis (*µ*_*AA*_ = *µ*_*Aa*_ *= µ*_*aa*_) using a χ^2^ test with two degrees of freedom. The alternative hypothesis was that genotype effects were not all equal. False Discovery Rate (FDR) was applied (R package *qvalue*^32^) to correct for multiple testing instead of stringent Bonferroni correction. FDR <= 0.10 was chosen as significance threshold to detect SNPs associated with low heritable female fertility traits. The same significance threshold was also chosen in previous study of detecting dominance effects^31^. Second, the SNPs with significant associations were then tested for the mode of gene action (additive and dominance). The additive effects^11^ were calculated as *a* = (*µ*_*aa*_ − *µ*_*AA*_*)/*2 and dominance effects^11^ were calculated as *d* = *µ*_*Aa*_ − (*µ*_*aa*_ + *µ*_*AA*_*)/2*. Testing of the significance was carried out using a *t* test against a null hypothesis of no additive and dominance effect. Additive and dominance effects were declared significant when they were different from zero (*P*-value < 0.01). Under the assumption of Hardy-Weinberg equilibrium, the additive variance was calculated as *2p(1 − p)[a* + *(1 − 2p)d]*^*2*^ and the dominance variance was calculated as *[2p(1 − p)d]*^2^, with p being the frequency of allele a.

### Simulation of phenotypes

Simulations were carried out to test the power and precision and when detecting dominance effects, using the available genotypes from chromosome 25 (arbitrarily selected) and pedigree from 3,040 genotyped heifers. The simulated phenotypes included polygenic effects, 3 QTL effects (sum of additive effects and dominance effects), and residuals. The polygenic effects were simulated from the oldest to the youngest animal in the pedigree. The polygenic effects of founder animals were sampled from the standard normal distribution as ${a}^{F} \sim {\rm{N}}(0,1)$. Polygenic effects of offspring with one known parent was sampled as ${a}^{o}\sim {\rm{N}}(\frac{a}{2},\,(\frac{3}{4}-\frac{F}{4}){\sigma }_{u}^{2})$, where $a$ was the polygenic effect for the known parent and *F* was the inbreeding coefficient of the known parent. Polygenic effects of offspring with two known parents was sampled as ${a}^{o}\sim N(\frac{{a}_{s}+{a}_{d}}{2},(\frac{1}{4}(1-{F}_{s})+\frac{1}{4}(1-{F}_{d})){\sigma }_{u}^{2})$, where ${a}_{s}$ and ${a}_{d}$ were the polygenic effects from the sire and dam, and ${F}_{s}$ and ${F}_{d}\,$were their inbreeding coefficients, and ${\sigma }_{u}^{2}$ was the polygenic additive genetic variance not explained by the QTL.

Three SNPs located at 10,109,903 bp, 20,058,762 bp and 30,039,582 bp on chromosome 25 were chosen as quantitative trait nucleotide (QTN), and with minor allele frequencies of 0.43, 0.19, and 0.09. These three QTN were chosen to represent high, medium, and low MAF. The additive genetic variances for each of these QTNs were simulated as either 5% or 10% of the polygenic genetic variance to represent the scenarios of the small and large QTL genetic variance. The dominance ratio *h* = *d/a* was simulated as −1, −0.5, 0.5, or 1. To mimic the low heritability of female fertility traits (<0.05) in Holstein populations, larger residuals were sampled as *e* $\sim {\rm{N}}(0,19)$ compared with polygenic effects sampled as ${a}^{F} \sim {\rm{N}}(0,1)$. In total, there were 10 simulation scenarios, which were the combinations of two different QTL genetic variances and five different ratios of dominance effects and additive effects. Each scenario was run 100 times. The power of simulations was calculated by the ratio of the detected QTL with significant dominance effects and the total number of simulated QTL (300), and the precision of simulations was calculated by the average of absolute distance (in Mb) between the positions of the detected (the most significant SNP) and the simulated QTN.

## Results

### Descriptive statistics

The descriptive statistics of female fertility traits for all animals with phenotypes (714,759 heifers and 905,447 cows) and for genotyped subset of animals (3,040 heifers and 4,483 cows) are presented in Table 1. In general, heifers had lower IFL and NIN than cows. Besides, genotyped animals had poorer fertility performance but larger standard deviation compared with all animals with phenotypes: IFLh was 5.6 days longer, NINSh was 0.056 times less, ICF was 2.0 days longer, IFLc was 1.4 days longer, and NINSc was 0.041 times less.

**Table 1:** Descriptive statistics for female fertility traits.

Traits^a^	All^b^			Genotyped^c^		
	N	Mean	SD	N	Mean	SD
IFLh	713,453	29.7	50.7	3,040	35.3	60.6
NINSh	711,805	1.862	1.243	3,033	1.918	1.372
ICF	900,418	49.2	64.5	4,479	51.2	67.1
IFLc	897,710	77.5	35.8	4,483	78.9	35.5
NINSc	895,807	2.336	1.522	4,461	2.377	1.595

^a^IFLh = days from the first to last insemination in heifers; NINSh = number of inseminations in heifers; ICF = days from calving to first insemination; IFLc = days from the first to last insemination in cows; NINSc = number of inseminations in cows.

^b^All = all animals with phenotypes.

^c^Genotyped = genotyped animals.

### Additive and dominance variance

The estimated additive and dominance genetic variances for heifer traits are summarized in Table 2. For cow traits, the genomic BLUP models failed to converge. For heifer traits, both proportions of phenotypic variances explained by additive and dominance variances were generally low (below 0.07). Besides, the dominance genetic variance was larger than the additive genetic variance. For example, for IFLh, the dominance genetic variance was 240 days^2^, while the additive genetic variance was 210 days^2^. Furthermore, large standard errors were observed for both additive and dominance genetic variances.

**Table 2:** Estimated additive and dominance genetic variance and the according proportions of phenotypic variance explained.

Trait^a^	Variance components			Variance explained^e^	
	Additive^b^	Dominance^c^	Residual^d^	Additive	Dominance
IFLh	210.1 (94.5)	240.6 (185.8)	3125.8 (162.1)	0.06	0.07
NINSh	0.05 (0.04)	0.08 (0.09)	1.71 (0.08)	0.03	0.04

^a^IFLh = days from the first to last insemination in heifers; NINSh = number of inseminations in heifers.

^b^Additive = additive genetic variance.

^c^Dominance = dominance genetic variance.

^d^Residual = residual variance.

^e^Variance explained = proportions of the phenotypic variance explained by the genetic variance.

### QTL with additive effect

**Figure 1:**
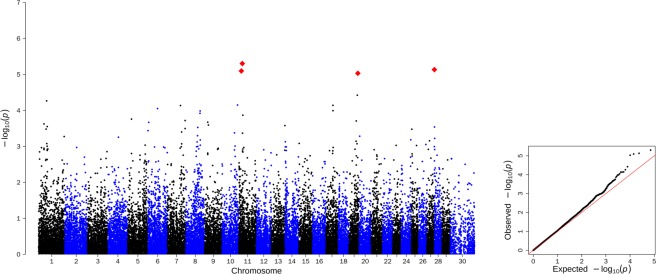
Left panel: Manhattan plot of genome-wide −log_10_(P-values) for SNP effects for IFLh (days from the first to last insemination in heifers). Significant SNPs (false discovery rate <= 0.10) are represented by red diamonds and these SNPs show significance for both additive and dominance effects (detailed information in Table 3). Right panel: QQ plot of genome-wide −log_10_(P-values) for SNP effects for IFLh.

A summary of significant SNPs is presented in Table 3. The Manhattan plot for SNP with additive effects for IFLh is shown in Fig. 1. The Manhattan plots for other traits are in Supplement File S1. Five SNPs located on chromosome 9, 11, 19 and 28 were associated with female fertility in Danish Holsteins, four SNPs for IFLh, one for IFLc. No significantly associated SNP was identified for ICF, NINSh or NINSc. On chromosome 9, there was one SNP (rs29018921) at 87,888,653 bp with a significant additive effect (*P*-value = 8.86E-6) for IFLc. On chromosome 11, there were two SNPs with significant additive effects for IFLh. They were rs109730886 located at 15,201,728 bp (*P*-value = 1.42E-6), and rs41592172 located at 21,081,322 bp (*P*-value = 8.33E-7). On chromosome 19, rs109922122 located at 55,191,677 bp had a significant additive effect (*P*-value = 2.01E-5) for IFLh. On chromosome 28, SNP rs109743925 located at 177,494 bp had a significant additive effect (*P*-value = 3.14E-6) for IFLh.

**Table 3:** Summary of significant SNPs for female fertility traits in Danish Holstein cattle.

Chromosome	Trait^a^	Position	SNP	MAF^b^	SNP effect		*P*-value^c^		Variance explained^d^	
					Additive	Dominance	Additive	Dominance	Additive	Dominance
11	IFLh	15,201,728	rs109730886	0.11	−25.81	−25.15	1.42E-06	1.38E-05	1.95E-03	0.01
11	IFLh	21,081,322	rs41592172	0.23	13.23	−9.66	8.33E-07	1.93E-03	0.01	3.30E-03
19	IFLh	55,191,677	rs109922122	0.43	−7.44	−6.5	2.01E-05	3.10E-03	0.01	2.86E-03
28	IFLh	177,494	rs109743925	0.13	−23.33	−18.16	3.14E-06	7.72E-04	0.01	4.75E-03
9	IFLc	87,888,653	rs29018921	0.11	7.31	−9.15	8.86E-06	2.36E-07	5.57E-06	0.01

^a^IFLh = days from the first to last insemination in heifers; IFLc = days from the first to last insemination in cows.

^b^MAF = Minor allele frequency.

^c^*P*-value = *P*-value from a chi-square test.

^d^Variance explained = proportions of the phenotypic variance explained by the SNP.

### QTL with dominance effect

The SNPs with significant additive genetic effects also had significant dominance genetic effects (Table 3). The Manhattan plot for SNP with dominance effects for IFLh is shown in Fig. 1. In general, dominance effects were less significant than additive effects. For example, the rs109922122 located at 55,191,677 bp on chromosome19 had a *P*-value of 2.01E-5 for additive genetic effect, but only had a *P*-value of 0.003 for dominance effect. The estimated dominance ratios *h* for five QTLs ranged from −1.25 to 0.97.

### Simulation

**Figure 2:**
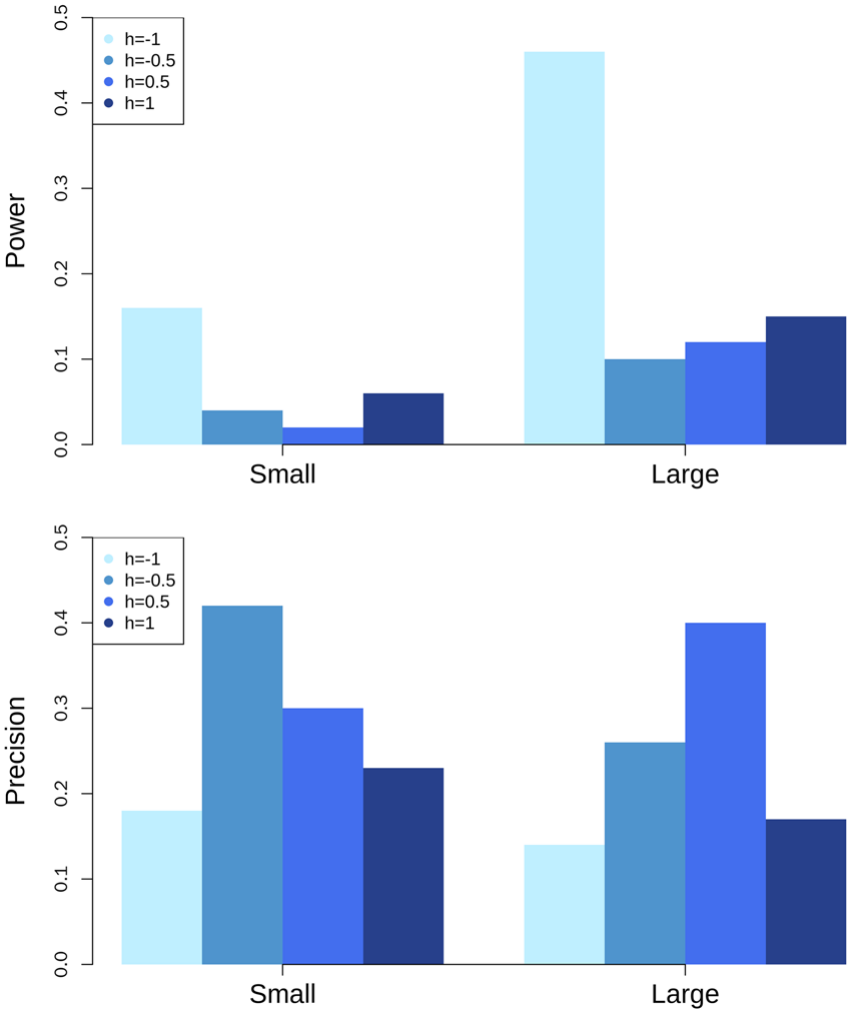
The power (upper panel) and precision (lower panel) for detecting dominance effects in simulations. The ratio of dominance over additive effect is denoted as h. Y axis shows the proportion of quantitative trait nucleotide (QTN) detected for power (upper panel) and precision in Mb for precision (lower panel); X axis represents the small and large QTN genetic variance. Small and Large represent that each of these QTN genetic variances is simulated as 5% or 10% of the polygenic genetic variance.

The power and precision of the QTL mapping of dominance effects are shown in Fig. 2. In the scenario with small QTL variance, maximal power (0.16) was achieved with *h = *−1, while the lowest power (0.02) was achieved with *h = *0.5. In the scenario with large QTL variance, the power to detect dominance effects was larger compared with that in the scenario of small QTL variance. Maximal power (0.46) was achieved when *h = *−1, while the lowest power (0.1) was achieved when *h = *−0.5.

In the scenario with small QTL variance, the highest precision (0.18 Mb) was achieved when *h* was −1, while the lowest precision (0.42) was achieved when the *h* was −0.5. In scenarios with large QTL variance, the precision was in general higher than that in the scenarios with small QTL genetic variance except when *h* was 0.5. The highest precision (0.14 Mb) was achieved when the *h* was −1, while the lowest precision (0.4) was achieved when the *h* was 0.5.

## Discussion

The genetic architectures of dominance effect for female fertility traits in Danish Holstein were investigated in this study. Several QTLs discovered for dominance effects provided novel variants for further investigation. Besides, our *post-hoc* simulations using existed genotype dataset provided a justification for the power and precision to detect dominance effects with current datasets.

The substantial dominance genetic variance in heifers indicates that dominance effects are important for genetic architecture of female fertility traits, at least for heifers. For example, both IFLh and NINSh had larger dominance genetic variances than additive genetic variances. A previous study demonstrated that the dominance genetic variance was moderate smaller than additive genetic variance in real data for number of teats, back fat, and average daily gain in pigs^33^. Other possible reasons for larger dominance genetic variance could be due to recessive lethal alleles. It has been reported that recessive lethal alleles play an important role on fertility traits in modern dairy cattle^17,34^. At low frequencies of the recessive allele, the recessive lethal would contribute mostly to dominance genetic variance in fertility traits. However, the large standard errors for both additive and dominance genetic variance hinder us to make a fair comparison between these two estimates. Low heritability and limited numbers of genotyped heifers could make the accurate estimation of variance components challenging^14^.

Additive and dominance effects were both significant in each of the identified QTL. This probably indicates that both additive and dominance effects jointly affect the genetic architecture of female fertility traits. This could also be that additive and dominance effects are not statistically independent. In our study, the significances of dominance effects were in general smaller than those of additive effects. This probably indicates that the power to detect dominance effects was lower than the power to detect additive effects. This might be explained by that the success of detecting additive effects depends on the presence of high linkage disequilibrium (LD) r^2^, where r is the correlation between the observed SNP and the causal variant. In comparison, the dependence on high LD between observed SNPs and causal variants is much stronger (r^4^) when detecting dominance effects^34^.

A previous GWAS used data from 3,475 Nordic Holstein bulls genotyped with the BovineSNP50 Beadchip to discover additive associations between SNPs and eight female fertility-related traits^35^. In their study, a SNP located at 87,568,944 bp on chromosome 9, in the vicinity of rs29018921 detected in this study, was reported to be associated with IFLc (*p* = 1.34 × 10^−8^). The rs29018921 is located in the 3′ untranslated region of gene peptidylprolyl isomerase like 4 (*PPIL4*). This gene was shown to be associated with milk production traits in a selection signature study in US Holstein cattle^36^. However, no female fertility-related functions of this gene are known.

This study also showed that rs109730886 and rs41592172 on chromosome11, rs109922122 on chromosome 19, and rs109743925 on chromosome 28 were associated with IFLh. However, these regions were not reported in previous studies for Nordic Holsteins^35,37,38^. The genes near these SNPs do not have known female fertility-related functions. The discrepancies with previous studies might indicate that they are novel genes for female fertility traits, or they are false discoveries due to low number of genotyped records in our dataset. However, these newly identified associations might be served as candidate SNPs for future investigations.

The simulations estimated the power and precision based on the current heritability, genotyping data and sample size. Power increased with the absolute value of degree of dominance (*h)* increased. For example, in the scenario with low QTL genetic variance, power increased from 0.04 to 0.16 when *h* changed from −0.5 to −1; in the scenario with high QTL genetic variance, power increased from 0.1 to 0.46 when *h* changed from −0.5 to −1. This clearly demonstrated that if there was more weight on dominance in the mode of gene action, more detection power will be achieved. Overall, it was difficult to discover dominance effects if the dominance genetic variance was low as a result of low QTL variance. In the scenario with low QTL variance, the power to detect dominance effects was only 0.02 when *h* was 0.5. The simulations also showed that the current study did not have high power to detect dominance effects, probably due to the low QTL genetic variance. The effect of sample size was not explored in this study. However, the limited sample size might have led to inaccurate estimates of additive and dominance genetic variances. For example, limited QTL detection power for additive genetic effects of female fertility was observed in the GWAS for Chinese Holsteins^37^, which had a similar reference population size (4,388 cows and 167 bulls). Thus, for female fertility traits, more animals need to be genotyped for future studies. Besides, we need to pay attention to the possible biased selection of cows to genotype.

Female fertility performance of heifers was superior to that of cows in Danish Holsteins, which was also observed in a previous study^5^. Furthermore, the genotyped animals had poorer fertility performance compared with all animals having phenotypes, but with a large standard deviation. The reason might be that genotyped cows were pre-selected for higher milk yield, which had negative genetic correlation with female fertility.

## Conclusions

Dominance genetic variances were larger than additive genetic variances in heifers, but with large standard errors. Five QTL located on chromosome 9, 11, 19 and 28 were detected for female fertility in Danish Holsteins, which were significant for both additive and dominance effects. Simulations indicated that the current sample size had limited power to detect dominance effects for female fertility. More females need to be genotyped to map genetic variants with dominance effects for female fertility.

## Supplementary information

Supplement File S1.

## Data Availability

Data supporting this paper were obtained from the commercial dairy farms in Nordic Countries. The phenotype and genotype data are available only upon agreement with commercial breeding organizations and should be requested directly from the breeding organizations.
