# Supplementary material for: Genome-wide association mapping for dominance effects in female fertility using real and simulated data from Danish Holstein cattle
Source: Sci Rep. 2020 Feb 19;10:2953. doi: 10.1038/s41598-020-59788-5 (PMC7031268; doi:10.1038/s41598-020-59788-5)
Supplement: Supplementary file 1 — Supplement File S1. [file 41598_2020_59788_MOESM1_ESM.pdf]

Genome-wide association mapping for dominance effects in female fertility using real and simulated data from Danish Holstein cattle

Xiaowei Mao,[1234]* Goutam Sahana,[3] Anna Maria Johansson,[4] Aoxing Liu,[35]
Ahmed Ismael,[346] Peter Løvendahl,[3] Dirk-Jan De Koning,[4] Bernt Guldbrandtsen[3]

[1]Key Laboratory of Vertebrate Evolution and Human Origins, Institute of Vertebrate Paleontology and Paleoanthropology, Chinese Academy of Sciences, Beijing 100044, China
[2]CAS Center for Excellence in Life and Paleoenvironment, Beijing 100044, China
[3]Center for Quantitative Genetics and Genomics, Department of Molecular Biology and Genetics, Aarhus University, 8830, Tjele, Denmark
[4]Department of Animal Breeding and Genetics, Swedish University of Agricultural Sciences, 75007, Uppsala, Sweden
[5]Laboratory of Animal Genetics, Breeding and Reproduction, College of Animal Science and Technology, China Agricultural University, 100193, Beijing, China
[6]Scion, 49 Sala St, Rotorua New Zealand
*Corresponding author
Email addresses:
Xiaowei Mao: maoxiaowei@ivpp.ac.cn
Goutam Sahana: goutam.sahana@mbg.au.dk
Anna Maria Johansson: anna.johansson@slu.se
Aoxing Liu: aoxing.liu@mbg.au.dk
Ahmed Ismael Sayed Ismael: ahmed.ismael@scionresearch.com
Peter Løvendahl: peter.lovendahl@mbg.au.dk

Dirk-Jan De Koning: dj.de-koning@slu.se

Bernt Guldbrandtsen: bernt.guldbrandtsen@mbg.au.dk

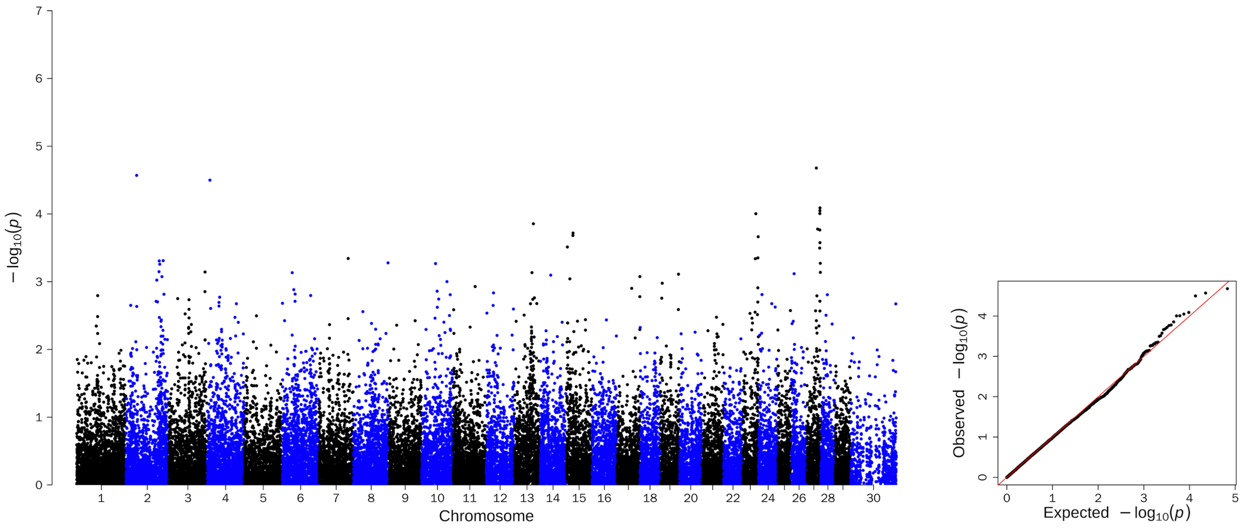

Figure S1. Manhattan plot (Left) and QQ plot (Right) of genome-wide −log10(P-values) for SNP effects for ICF (days from calving to first insemination). There are no significant SNPs with false discovery rate <= 0.10 for ICF.

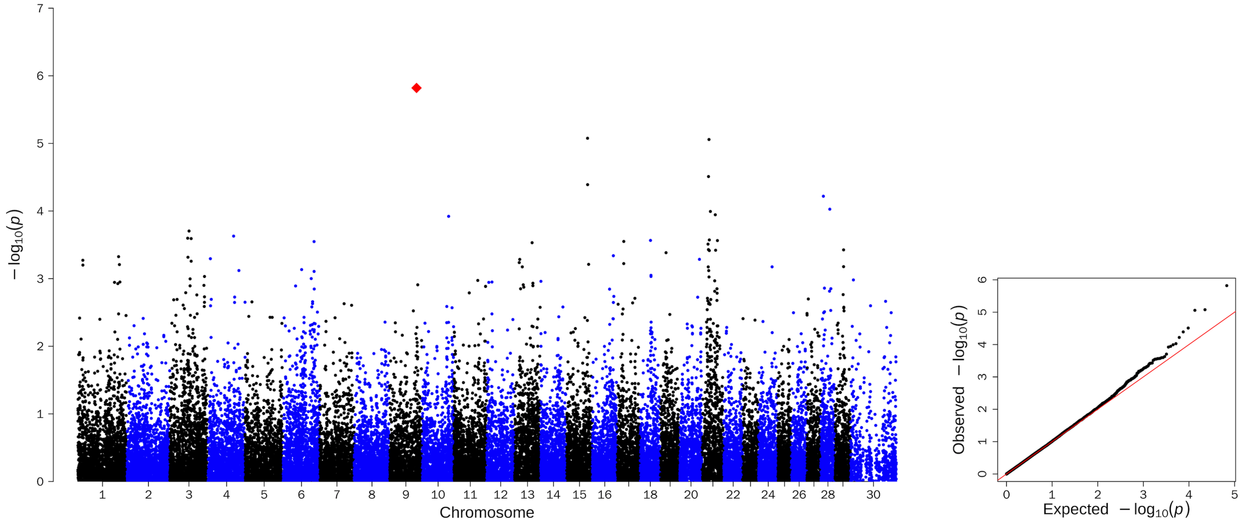

Figure S2. Manhattan plot (Left) and QQ plot (Right) of genome-wide −log10(P-values) for SNP effects for IFLc (days from the first to last insemination for cows). Significant SNPs (false discovery rate <= 0.10) are represented by red diamonds and one SNP show significance for both additive and dominance effects (detailed information in Table 3).

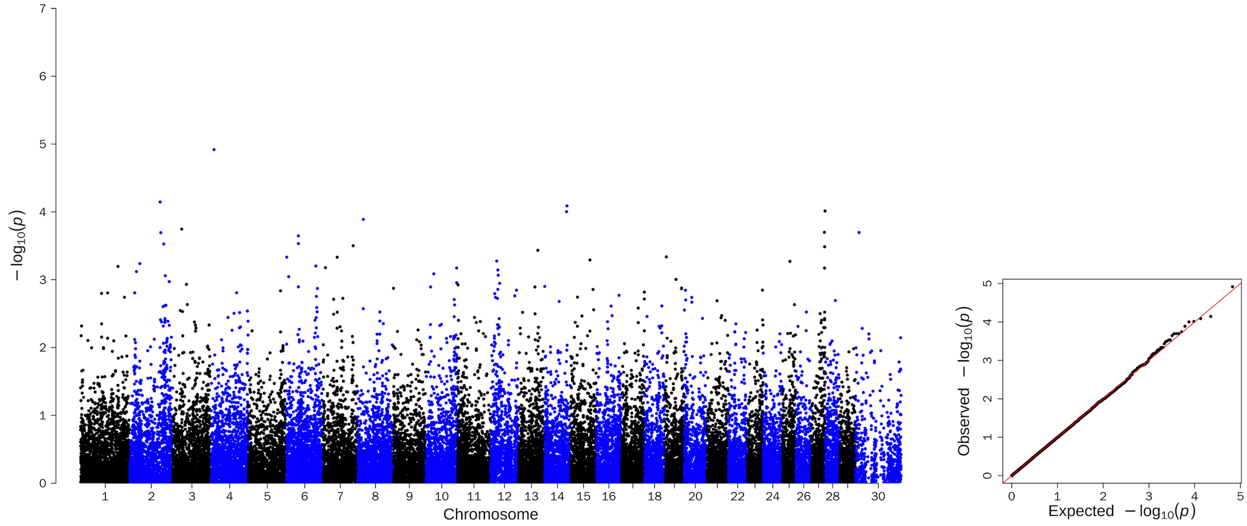

Figure S3. Manhattan plot (Left) and QQ plot (Right) of genome-wide −log10(P-values) for SNP effects for NINSc (number of inseminations for cows). There are no significant SNPs with false discovery rate <= 0.10 for NINSc.

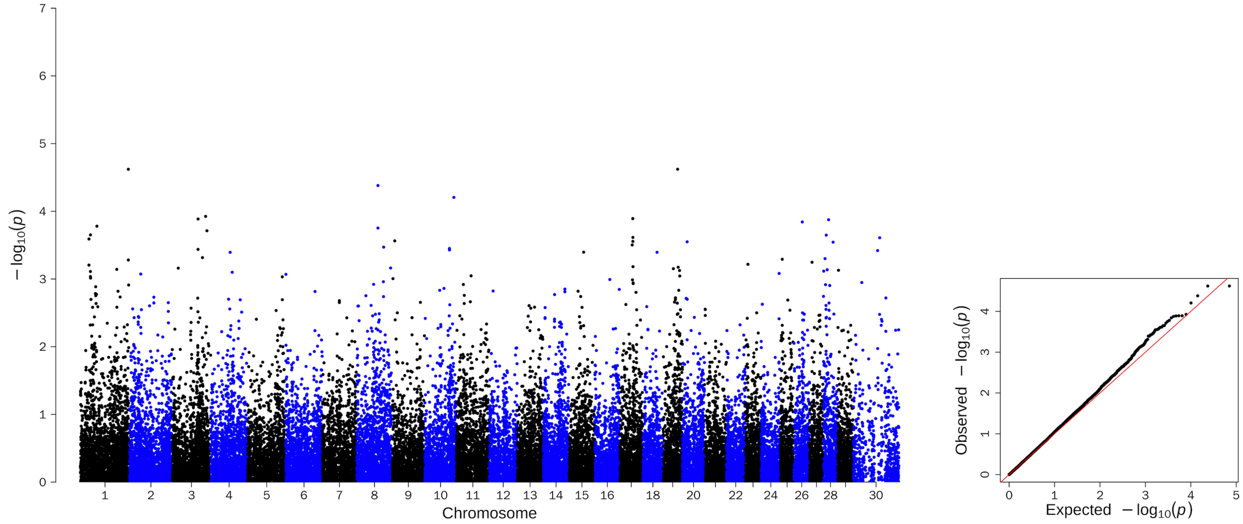

Figure S4. Manhattan plot (Left) and QQ plot (Right) of genome-wide −log10(P-values) for SNP effects for NINSh (number of inseminations for heifers). There are no significant SNPs with false discovery rate <= 0.10 for NINSh.
